# Collaborative Approaches and Policy Opportunities for Accelerated Progress toward Effective Disease Prevention, Care, and Control: Using the Case of Poverty Diseases to Explore Universal Access to Affordable Health Care

**DOI:** 10.3389/fmed.2017.00130

**Published:** 2017-08-25

**Authors:** Samia Laokri

**Affiliations:** ^1^School of Public Health, Health Policy and Systems – International Health, Université Libre de Bruxelles, Brussels, Belgium; ^2^School of Public Health and Tropical Medicine, Global Community Health and Behavioral Sciences, Tulane University, New Orleans, LA, United States; ^3^Institute for Interdisciplinary Innovation in Healthcare (13h), Université Libre de Bruxelles, Brussels, Belgium

**Keywords:** universal health coverage, tuberculosis, use of knowledge, research-based guidance, evidence-based integrated care, public–private partnerships, translational research, system thinking

## Abstract

**Background:**

There is a massive global momentum to progress toward the sustainable development and universal health coverage goals. However, effective policies to health-care coverage can only emerge through high-quality services delivered to empowered care users by means of strong local health systems and a translational standpoint. Health policies aimed at removing user fees for a defined health-care package may fail at reaching desired results if not applied with system thinking.

**Method:**

Secondary data analysis of two country-based cost-of-illness studies was performed to gain knowledge in informed decision-making toward enhanced access to care in the context of resource-constraint settings. A scoping review was performed to map relevant experiences and evidence underpinning the defined research area, the economic burden of illness.

**Findings:**

Original studies reflected on catastrophic costs to patients because of care services use and related policy gaps. Poverty diseases such as tuberculosis (TB) may constitute prime examples to assess the extent of effective high-priority health-care coverage. Our findings suggest that a share of the economic burden of illness can be attributed to implementation failures of health programs and supply-side features, which may highly impair attainment of the global stated goals. We attempted to define and discuss a knowledge development framework for effective policy-making and foster system levers for integrated care.

**Discussion:**

Bottlenecks to effective policy persist and rely on interrelated patterns of health-care coverage. Health system performance and policy responsiveness have to do with collaborative work among all health stakeholders. Public–private mix strategies may play a role in lowering the economic burden of disease and solving some policy gaps. We reviewed possible added value and pitfalls of collaborative approaches to enhance dynamic local knowledge development and realize integration with the various health-care silos.

**Conclusion:**

Despite a large political commitment and mobilization efforts from funding, the global development goal of financial protection for health—newly adopted in TB control as no TB-affected household experiencing catastrophic expenditure—may remain aspirational. To enhance effective access to care for all, innovative opportunities in patient-centered and collaborative practices must be taken. Further research is greatly needed to optimize the use of locally relevant knowledge, networks, and technologies.

## Introduction

### Health for All—A Global Commitment in the Spotlight

Late 2015, the UN General Assembly adopted the sustainable development goals (SDGs) (1). Of those, goal 3 explicitly refers to “ensure healthy lives and promote well-being for all at all ages” and embraces to “achieve universal health coverage” (UHC) (2). During a panel hosted by the Chatham House in London on June 6, 2017, Tedros Adhanom, the freshly elected Director General at World Health Organization, and Amartya Sen, Professor of Economics at Harvard University and former Nobel Prize in Economics, joined their positions to raise awareness on the consequences for countries of not providing UHC with respect to poverty reduction and global development. There is indisputably a massive global momentum to progress toward UHC equitably and cost-effectively. Yet, its assessment represents significant political and technical challenges (3, 4). Among them, the financing strategy on which economists estimated global returns on investment in equity and universal coverage at more than ten times their costs (5). On the one hand, there is still a scarcity of national evidence on effective policies for health coverage. On the other hand, where evidence is available, research findings are too little used. Moreover, evidence that is not disseminated or used can be seen as a source of inefficiency (6). How to translate the powerful concepts of UHC into local actions place a rationale for locally relevant knowledge development and data use for effective decision-making.

Building well-functioning systems to maintain or reach sustainable UHC require constant attention, a long-term development process (7) and difficult trade-offs to make right decisions (8). Today, few countries escape these questions, whether it is for planning, initiation, and development of new programs, expansion and funding of already established programs, or attainment of cross-cutting goals such as equity and efficiency to meet the growing demands of our societies (9). Along with sustainability, a translational perspective toward effective policies is needed (10). Moreover, health policies aimed at removing user fees for a defined health-care package (will continue to) fail at reaching the desired results if not applied with system thinking. System thinking in public health became widely recognized as an approach to reflect on complexity and systems strengthening (11, 12). Much remains to be done to effectively reduce the global burden of disease. Nevertheless, despite considerable progress, many countries experience scarce or wasted resources and do not deliver primary and secondary care services as targeted (13). To date, too many people still face catastrophic health expenditure every year. Approximately 150 million people around the globe with two-thirds forced into poverty as a result of health spending (14).

### A Focus on Poverty-Related Diseases

In many ways, poverty diseases would constitute prime cases to better understand the efforts made to, and assess the extent of, effective coverage for high-priority health-care services.

For instance, tuberculosis (TB) could have been a disease of the past since the discovery of the *Mycobacterium tuberculosis* by microbiologist Robert Koch dates from 1882 (15). TB however remains one of the worst health scourges despite an ever growing global commitment to fight and hopefully eradicate the disease burden. Actually, TB even became the world’s leading infectious killer, killing more people than HIV/AIDS (16). The case of TB control raised our interest for multiple reasons. To begin with, the End TB strategy recently reached a turning point in adopting a third ambitious goal on financial risk protection, as part of the United Nations SDGs (17). The global strategy aspires to eliminate all sufferings from catastrophic expenditure faced by TB-affected families and set an important milestone to be achieved by 2035. Besides, TB encloses a sub-sector in which innovation with respect to diagnosis, treatment, and prevention is still sought to effectively reach the global targets. Moreover, TB care services involve the first line of health-care providers and fully solicit the provision of quality primary health care (i.e., preventive, promotive, and curative care services), which fairly meets the UHC agenda. Additionally, poverty reduction, economic development, food security, or migration all relate to TB and the SDGs resolution.

In that way, the sound analysis of the patients’ care-seeking and care pathway that we propose represents an interesting opportunity to inform policy-making and national and international priorities. Obviously, using a single disease as predictor of health access and adherence barriers to care may not give a complete picture of the whole package of UHC services but can, in return, provide valuable evidence to move forward with evidence-based cost-effective and responsive policies.

### Exploring the Case of TB

Tuberculosis national control programs benefited from generous financing, mostly borne by a relatively small number of donors who support a directly observed treatment, short-course (DOTS). The TB care pathway is known to be particularly long and complex. As a result of these difficulties, we observe persisting inequities in access and catastrophic health expenditure (18, 19),–the latter, despite years of free-of-charge diagnosis and treatment under the global strategy.

As recalled above, financial protection for health remains a matter of concern that need to be tackled to make the global targets toward TB elimination by 2050 real. Freeing the world of the TB threat should involve to alleviate poverty and engage multisectoral actions (20). Further, challenges include to enhance translational research for TB elimination i.e., from “fundamental research to clinical, epidemiological, implementation, health system and social science research” (10) and consequently deliver valuable evidence. Lienhardt et al. stressed the role of both operational and fundamental research to align patients’ needs with the requirements of the development of new opportunities, especially for a timely identification of TB suspects as well as better responsive treatment regimens, vaccines, and care provision strategies. Such perspective relates to picture a continuum of prevention, care, and control services supplied in a holistic approach. For instance, new anti-TB drug regimens are expected to (i) ease and shorten the current lengthy first-line treatment of 6-month duration (when successful), which is based on a mixture of multiple drugs, (ii) restrain biomedical and other side effects, and (iii) eliminate the threat of discontinued drug regimens (mainly after the 2-month intensive treatment phase), as well as treatment failures and drug resistance (21). Furthermore, within the new era of global development, which set the WHO’s post-2015 End TB strategy in the frame of the SDGs, socio-economic determinants of TB and health systems strengthening stand as key issues (10, 22, 23).

## Aim and Method

Secondary data analysis of two country-based cost-of-illness studies was performed to gain knowledge in informed decision-making toward enhanced access to health care in resource-constraint settings.

**Figure 1:**
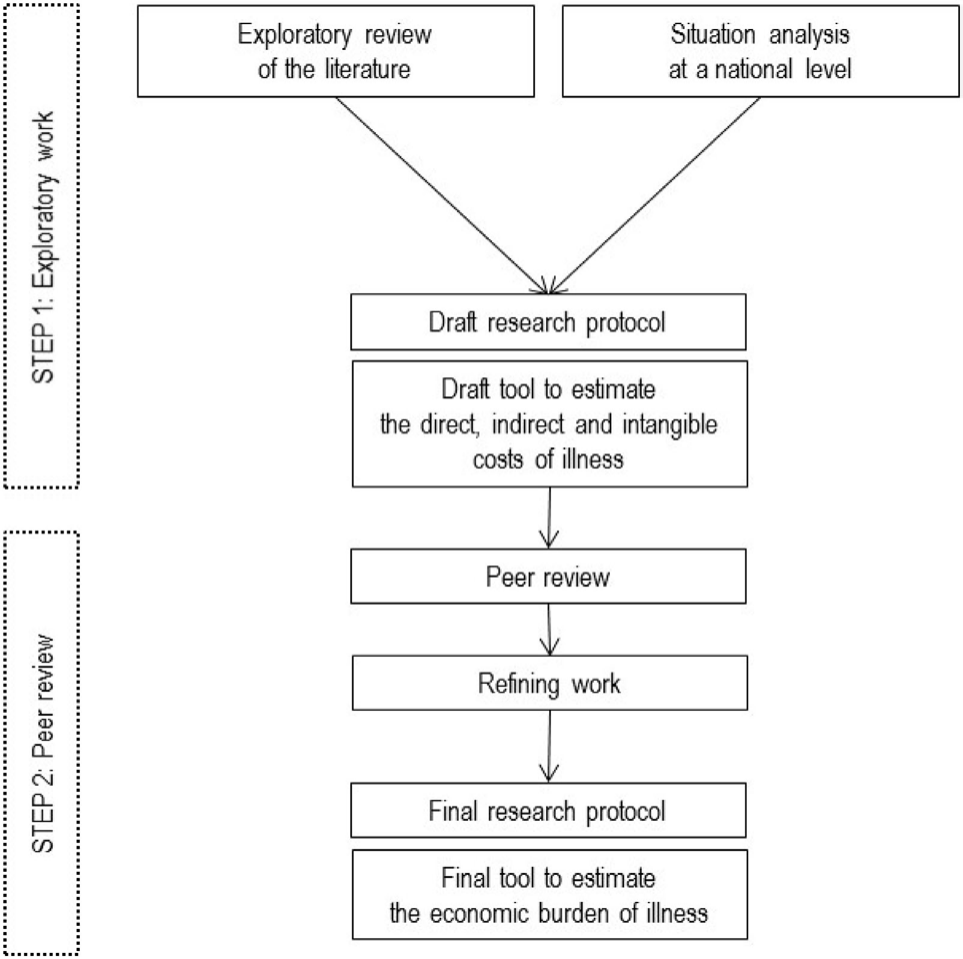
Research protocol development.

Original studies were conducted in sub-Saharan Africa, more precisely in Burkina Faso (24) and in Benin (25), using a single research protocol (26). A two-step process relying on state-of-the-art knowledge and peer review was designed to develop a cost-of-illness research protocol (Figure 1). First, baseline information was retrieved from an explanatory review of the literature, to determine knowledge gaps, and country situation analyses to determine the particular local needs. Second, a peer review process based on a multidisciplinary expertise was conducted in order to validate our study objectives and conceptual framework. This two-step process allowed us to adapt and refine standard study protocol and operationalization of research.

**Figure 2:**
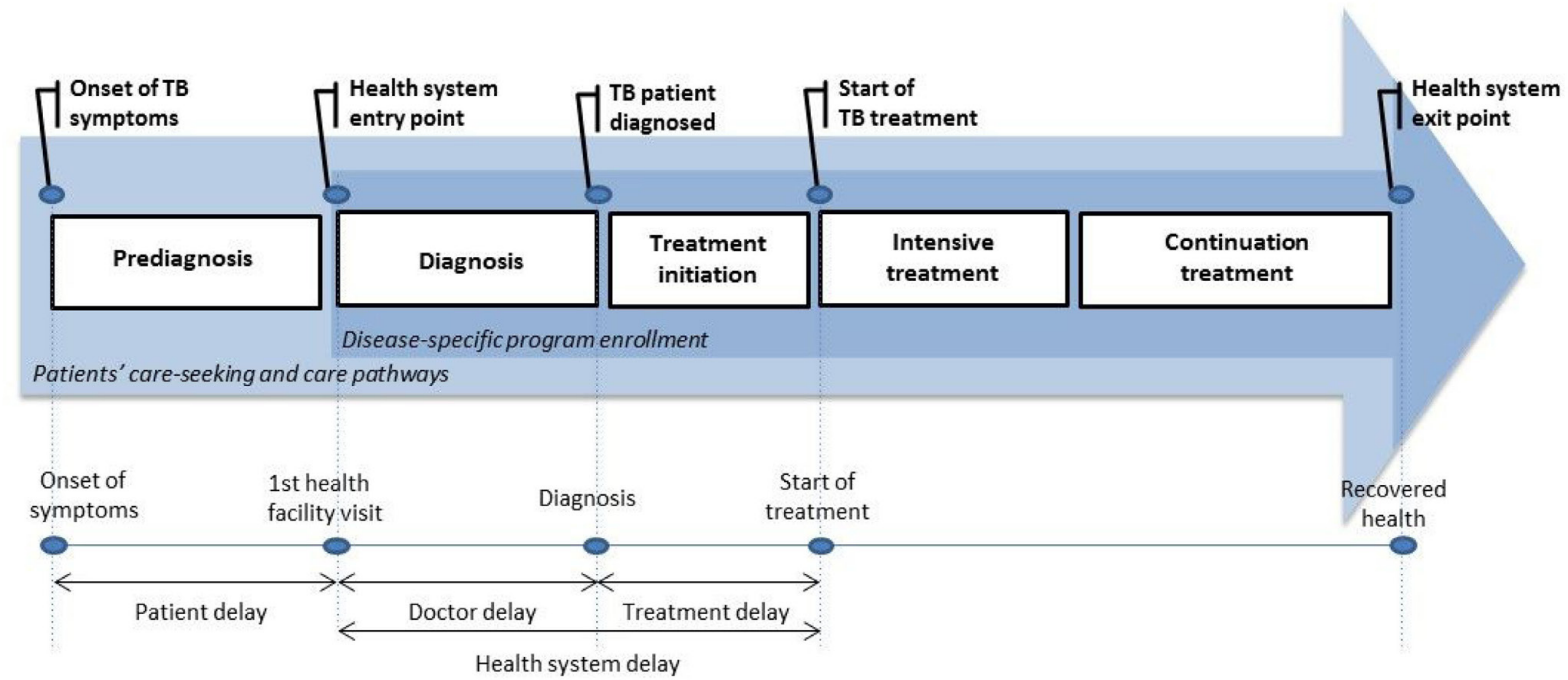
Cost-of-illness timespan: five key steps of the TB patients’ care pathway.

A cost-of-illness timeline was derived from our preparatory work, which included both a consultation with the key informants and national TB program experts involved in the research project and available literature on delays in TB treatment. According to available evidence, delayed diagnosis and treatment may differ widely between study settings (27). Therefore, beyond the usual “medical” stages related to diagnosis and treatment procedures, a context-oriented approach highlighted the importance of considering what could happen prior to diagnosis and immediately prior the initiation of treatment. We took the specific aspects of the DOTS into account to determine the key steps related to the successive periods of the care-seeking pathway. The first reference was to the onset of TB symptoms (e.g., prolonged cough). Thereafter, the key steps were the time of first contact with a public health-care provider, confirmation of the TB diagnosis, the beginning of intensive treatment, and finally, the beginning of continuation treatment. In total, we covered the entire care-seeking and care pathway from the onset of TB symptoms to the completion of treatment (Figure 2).

The data collection process exhaustively recorded all relevant events related to TB that had been already occurred when the study was initiated (retrospective design). Input data were gathered *via* patient survey to produce reliable estimates of the different components of costs and use of care services. This bottom-up study design provided an opportunity for patients to disclose expenses that were often neglected in contemporary investigations thus reducing possible cost underestimation. Similarly, precise estimates of time lost at work due to illness and resource mobilization strategies that were developed to cope with illness could have been collected. Countries specificity and methodology are fully described elsewhere (24, 25).

A secondary analysis of comparative study findings allowed us to highlight a series of policy gaps, producing a knowledge development framework. Subsequently, a research question was identified to explore potential added value of collaborative work approaches with respect to addressing the observed policy gaps. Then, a search strategy using combined keywords was performed through the usual means (electronic databases, reference lists of ancestor searching, and specific websites of organizations). Refining the scope, we focused on the specific role of public–private initiatives in reducing the economic burden of disease. A narrative integration of relevant references was performed. The scoping review was therefore implemented to map relevant experiences and evidence underpinning the defined research area.

## Findings

### Summary of Key Findings

In our two West African studies, three-quarters of the patients treated for TB under the DOTS faced catastrophic health expenditure. Catastrophe refers to health expenditure that placed excessive burdens on TB-affected families and is largely associated with adverse health outcomes and severe financial hardship for their members. Particularly, the incidence of catastrophic expenditure ranged from 38% in the upper income quintile of the study population to 94% in the lower income quintile in Benin, and the intensity of catastrophic expenditure ranged from 5 to 31%, respectively (28). In both studies, removing user fees for medical spending did not prevent the patients from financial distress due to access TB diagnosis and treatment services. While differences occurred with respect to the incidence and intensity rates of catastrophic expenditure across locations or wealth groups, the lack of financial protection remains a common dare for most programs regardless the environment.

It is not only about reaching increased TB control coverage but also about improving patient management, which is required to improve health-care delivery and progress toward UHC. Some authors had shaken the scientific community while saying that solutions to reduce time delays in care should be sought by the care providers (and not the patients); this includes patient delay (27). The findings from Burkina Faso provided new evidence in support of this hypothesis (18). Substantial hidden costs induced by apparent failures in delivery of health care for TB patients were highlighted. These were essentially due to structural and organizational flaws for almost half of the patients. Indeed, with respect to diagnosis only, “*39% of the patients had been charged, between first contact and the end of diagnosis, for sputum examinations, chest X-rays, other examinations or hospitalization (*…*), which amounted to US$ 8 per patient (*…*), to be paid within a fairly short period of time*.” As stated, the issues behind the reduction in time delays and expenses incurred at each stage of the patient’s care pathway remain important.

In sum, our findings indicate that the coverage of TB control programs might be greater than actual program outcomes. Substantial out-of-pocket payments for TB appeared to be generated by expenses falling outside of the free health-care package (e.g., pre-diagnosis, extra-consultations, non-medical spending), which may result in an underestimation of routine estimation of the overall economic burden of TB incurred by households. The series of remaining shortages and hidden failures shown in the health-care delivery system for TB patients highlighted windows of opportunities to facilitate the change in context. These called for data-driven decision-making that is suited to local necessities.

Besides the bleak picture depicted (29), our concern not only pertains to limited access due to excessive direct costs but also to indirect costs that took the form of days and income loss due to TB. In addition to lower resources while facing higher needs, the strategies that patients used to mobilize funds to cope with the substantial direct and indirect costs imposed strains on the families’ financial viability, through actions such as exhausting their savings, getting into debt, and even selling livestock and property (18). Damaging asset portfolios of disease-affected households in the long run, the riskier coping strategies result in a public health threat.

### Knowledge Development Framework and Informed Decision-Making—The Cornerstones of Strong and Integrated Health Policies

Our study observations were congruent with the literature in the field (19, 30, 31). In various settings, key findings confirmed substantial direct and indirect costs associated with TB. Although the national programs delivered free diagnosis and treatment, the TB control strategy tend to remain unaffordable and inaccessible for TB-affected households living with limited resources (22). This viewpoint features a rather tunnel vision of the previous global strategy with respect to reaching financial protection *via* user fee exemptions for biomedical matters (32). Removal of those fees did allow a major step forward in access to TB control services overseas, but this was no longer sufficient to eliminate TB as a public health problem (21). Therefore, the post-2015 global strategy enlarged his scope to cover adverse economic effects associated with seeking and receiving TB services (16). This establishes the need to assess and monitor catastrophic expenditure due to TB, its drivers, and consequences.

**Figure 3:**
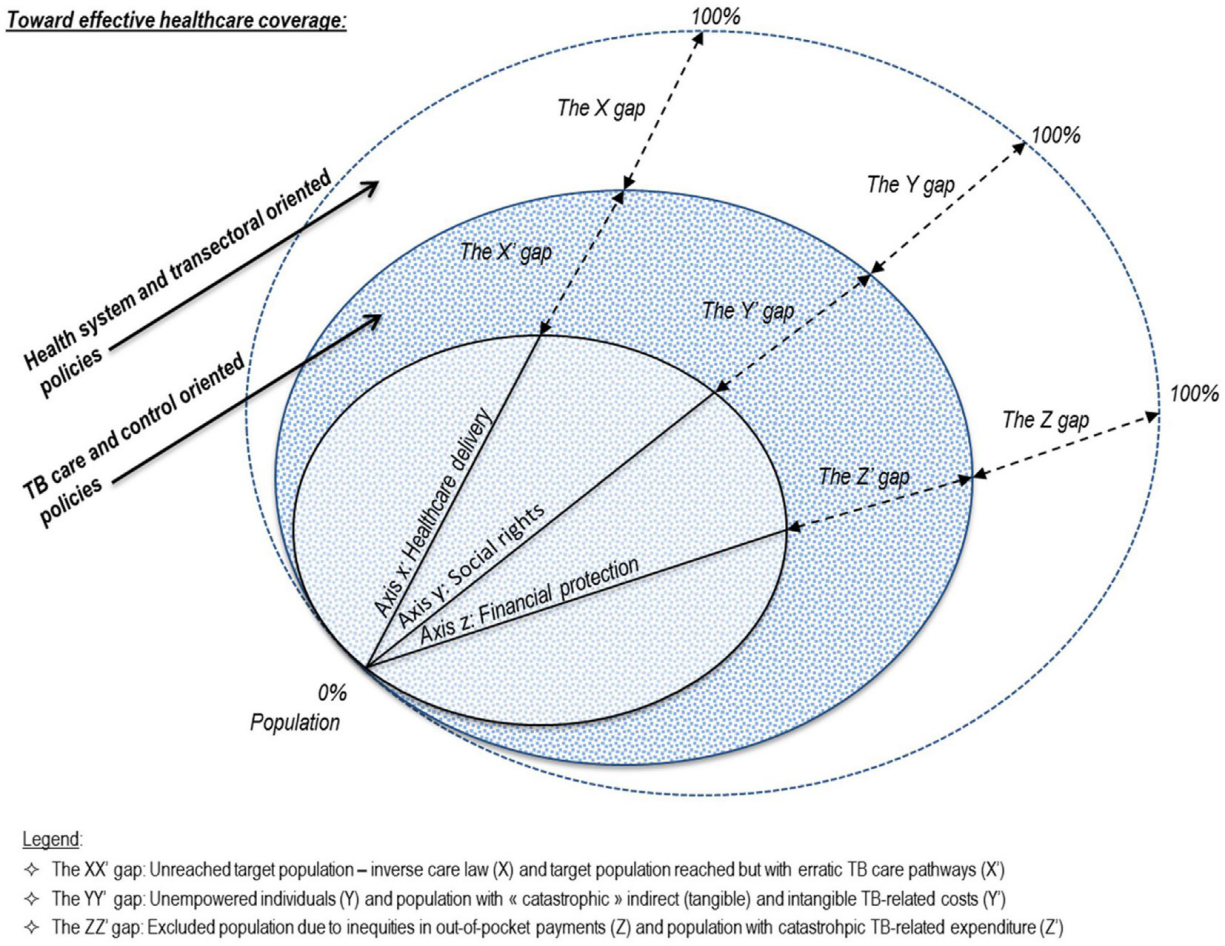
The complex path toward effective TB prevention, care, and control coverage.

To feed the global agenda, we investigated a series of potential shortcomings to effective policy toward TB elimination and scaling-up areas. We screened bottlenecks on access to care, equity as well as programmatic, implementation, and managerial or behavioral aspects. We attempted an analytical approach to effective TB prevention, care, and control coverage. Based on three closely interrelated patterns, Figure 3 portrays a complex path where:

- Health-care delivery performance—axis *x* represents the proportion of the population with access to timely and good-quality TB services according to their needs.
- Social rights attainment—axis *y* provides the proportion of the population whose basic individual necessities and capabilities needed are met in order to seek and receive care services effectively.
- Financial risk protection—axis *z* refers to the proportion of the population with access to TB services without being distressed or impoverished as a consequence of using TB services.

There is no single path to effective coverage. Our frame echoes the coverage dimensions of the WHO’s UHC cube representing population, services, and direct costs covered (33) and its permutation in the context of TB (32). On each axis, a “double policy gap” intends to highlight both the remaining path to extending TB control strategy and efforts to obtain effective coverage for those already reached by the health program. From our findings, the determination of programmatic flaws and scaling-up coverage needs aim to elude adverse effects incurred by TB-affected households. Those were inherent consequences of erratic patients’ pathways, inappropriate use of multiple-care services, supply side weaknesses, and delayed or incomplete recovery of health.

Similar to many others, we focused on patients who were already enrolled in the DOTS. We remain able to make the following three observations. First, as there are TB-affected individuals whose illness is undetected and untreated, there is still a target subpopulation that is not reached by the national TB control programs (NTPs). Reasons for the so-called X gap may include limited availability of public health care and TB services, poor case management of comorbidities, inequities in TB knowledge, or a mismatch between supply and demand. Poor health systems organization also relates to the absence of coordination bodies, surveillance studies, and joint actions between various health programs. Indeed, despite a “one-stop TB-HIV” approach recommended by the WHO, TB, and HIV programs often operate independently (34). A similar or even more severe observation can be made for coordination with other programs. Second, there are still sick individuals who suffer from a variety of ailments that make them unable to exercise their rights. Subsequently, there is a non-empowered target subpopulation without access to the needed services, the Y gap. Third, when financial barriers, even to high-priority services such as free diagnosis, persist due to lack of prepayment and pooled schemes, there is a target subpopulation unable to afford TB-related services. The absence of a compulsory financial protection scheme (no prepayment or cross subsidies in the population) results in health system failures, the Z gap. These shortages establish three “primary” policy gaps, which relate to the integration of health programs into health systems and transversal policies.

Analysis of the inner circles highlighted the need for additional policy responses, mostly located at a programmatic level. Drawing on our West African studies findings, we extended this argument further. In addition to the primary policy gaps, there are hidden gaps that plague the fulfillment of various patients’ needs. Indeed, we identified several weaknesses within the health-care delivery system for TB patients enrolled in the DOTS. Addressing these weaknesses may be a strategic step toward reducing the primary policy gaps. For each dimension, we established a “side” gap. First, we revealed a quality issue in the management of patients, which may have contributed to erratic care pathways. The X′ gap refers thus to a subpopulation that has been reached by NTP networks but for which suboptimal care coordination and patient management was provided (e.g., provider delays, redundant visits). The findings suggested that the current strategy lacks patient-centered care, a context-oriented approach, and systemic vision. This highlights the need to consider the extent to which the disease-specific programs deliver responsiveness, relevance, effectiveness, and efficiency in their activities and implementation processes. Second, an additional side gap that emerged refers to the adverse effects of indirect and coping costs. To begin, there is the spiral of poverty embodied by labor and income losses and recourse to impoverishing coping strategies such as indebtedness. Furthermore, there is the issue of overburdened individuals and social exclusion effects potentially induced by illness stigma, with a concrete risk of being excluded from services (public health-care services) and participation. This policy gap therefore encompasses costs of a very different nature, which range from tangible costs, such as income loss and charges related to indebtedness, to intangible costs that may have disruptive effects on households and likely affect human rights and health equity. This Y′ gap thus defines a subpopulation unable to elude catastrophic tangible indirect and intangible costs of illness. Third and finally, we recall the importance of considering overall out-of-pocket expenses (medical/non-medical, and pre-/post-diagnosis) incurred by the patients, and their magnitude in relation to households’ resources that need to be mobilized. The so-called Z′ gap isolates a subpopulation incurring catastrophic expenditure as a consequence of the use of health care and TB services.

### What May Drive the Economic Burden of Illness?—Translating Research Findings into Policy Practice

Addressing the above policy gaps concurrently should remain a major global health issue. Translating evidence into enhanced patients’ outcomes rely on efforts from the scientific community to facilitate this complex process. Applying our framework, a simulation on our case study confirmed a potential room for improvements in terms of financial gain for beneficiaries associated with concurrent effective implementation and patient-centered and comprehensive care delivery (35).

Particularly, erratic care-seeking pathways generated inconvenient but potentially remediable health-care expenses for TB. The breakdown analysis of the nature and volume of TB-related direct out-of-pocket costs allowed us to identify a series of areas of progress.

Hidden and potentially provider-induced medical costs (e.g., the systematic prescription of chest X-rays for TB diagnosis in some health facilities) were raised. We investigated whether and to what extent these hidden direct costs may have been reduced *via* improved patient management schemes (i.e., a patient-centered and context-oriented approach). Thus, we simulated the impact of the rationalization of delivery of care by the strict application of the national TB recommendations. Therefore, we made three sets of assumptions. As challenges imply on both the provider (supply) and patient (demand) sides, the following assumptions focused on both sides. First, we assumed that TB suspects were to be correctly informed regarding the symptoms distinguishing TB (e.g., 15 days of coughing) and had direct access to first-line health facilities to allow rapid referral for free TB diagnosis. Thereafter, according to the user fee exemptions for diagnosis, health-care costs incurred by TB patients during the diagnostic stage should remain affordable for them. Second, we assumed that the patients were to begin treatment as soon as their diagnosis was confirmed. One reason for this is that a patient diagnosed with TB is in the most infectious period of the disease at this stage. Indeed, patients with active pulmonary TB increase the risk of primary infection and reactivation of latent TB among their acquaintances. This means that neither treatment delay nor health-care costs had occurred between the diagnosis confirmation and the initiation of the anti-TB treatment. Third, we assumed that the DOTS was truly free of charge, effective, and sufficient to treat TB patients. Therefore, medical costs were required to tend toward 0 during both the intensive and continuation treatment stages. These assumptions may be considered strong, but the exercise was very informative, as it revealed a median financial gain (IQR) of 50.4% (26.3–67.0%) of overall direct cost by virtue of the removal of care delivery inadequacies and policy gaps.

**Figure 4:**
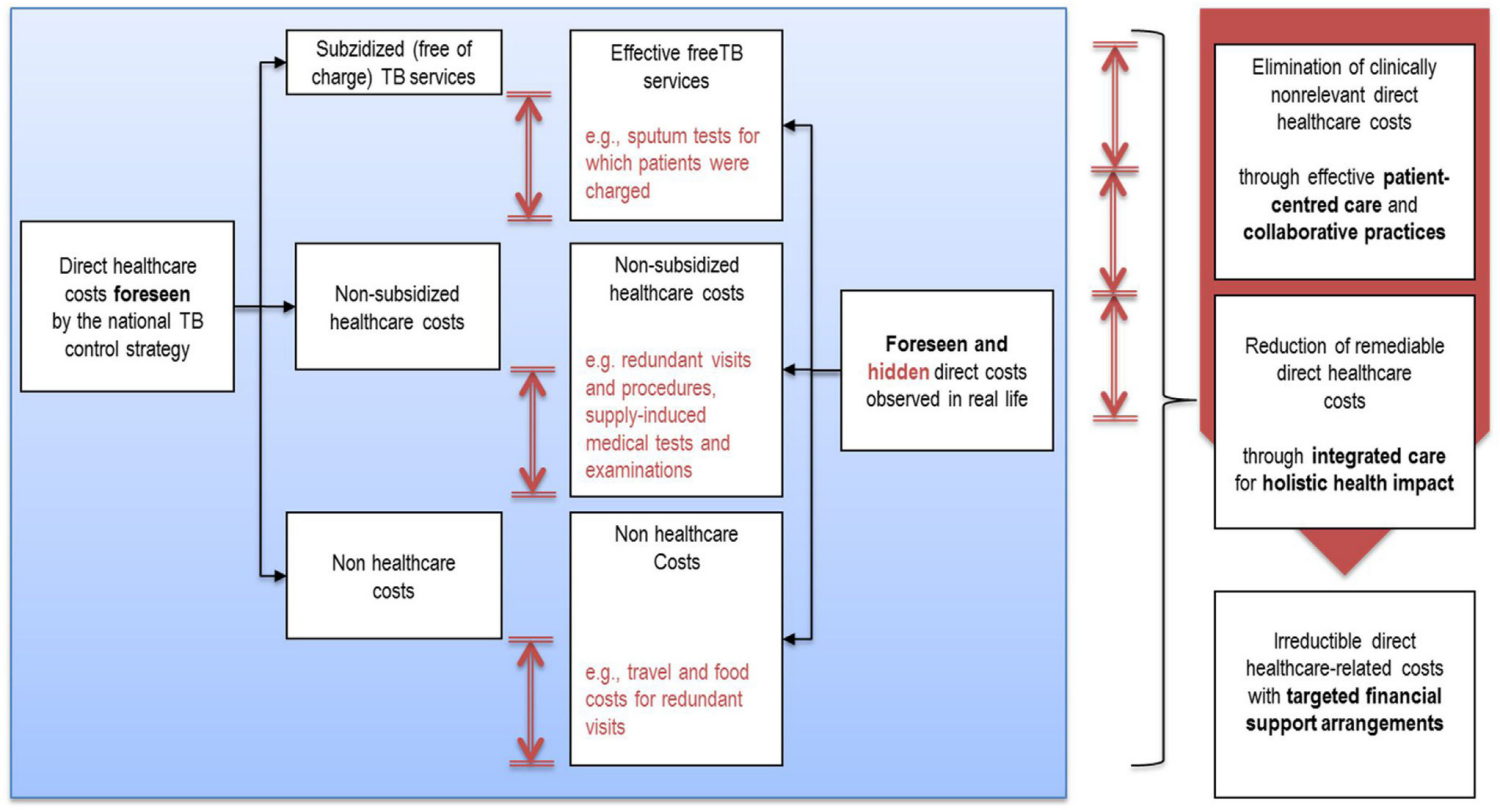
Substantial avoidable health-care costs due to apparent failures in the delivery care system.

If TB-related expenses could be halved, one can expect substantial progress in TB outcomes. Educating public health workers in the provision of a patient-centered approach and educating all stakeholders *via* a systemic approach could be relevant in reducing these avoidable hidden patients’ costs (Figure 4).

Consequently, the economic burden associated with illness ends up multidimensional. It embodies multiple risks and likely fuels the three “double” policy gaps. Moreover, it may start a vicious circle where the burdens of erratic care-seeking and care pathways tend to surge the risk of inappropriate care use and further increase the drug-resistant threat (35).

In view of the above, adopting a syndemic approach would be inspiring to better inform on the multidimensional aspects of effective TB coverage and related challenges with respect to case management of coinfected patients (36). Extrapolating from syndemics would suggest that patients’ care pathways are affected by the presence of comorbidities. For instance, multiple burdens such as TB-HIV/AIDS, diabetes, malnutrition (Vitamin D deficiency), or even tobacco smoking may adversely interact with each other and generate increased vulnerability and inequities (37). Both synergisms between TB and other health programs and syndemic interactions (including aspects related to household crowding, socio-economic constraints, and air pollution) are to be further established (38). Quoting the authors, “syndemic interactions play out over a life time and across the generations.”

## Discussion

### Collaborative Work for Affordable Primary Health Care for All—A Tentative Response to Mind the Stated Policy Gaps

Effective implementation of TB control activities depends not only on research-based guidance but also people’s performances to convert available inputs into outcomes. On the one hand, people are vehicles of valuable knowledge regarding care provision and consumption. On the other hand, policymakers tend to most value and use local data sources provided through personal contacts (39). Health-care services users and providers, as well as managers, financers, and knowledge agents, turned out to be strategic game changers to promote social justice (40). Beyond a recipe for response to TB and other diseases burden, an evidence base must be sought in resource-poor countries. Particularly, multi-stakeholders and people-centered approaches should be further studied as they appear as an emerging science based on human agency^1^ and people carrying system change (40).

Target populations often differ over time and from country to country or region to region. Nevertheless, most populations and certainly in low-income settings tend to seek care from biomedical care providers (public and private sectors) and trusted traditional healers. The private health sector can be defined as all non-state providers, which may cover a wide range of not-for-profit and for-profit actors including faith-based organizations, private health insurers, pharmacies, practitioners and hospitals, and traditional healers (41). Accordingly, care-seeking behaviors rely on the existence and use of concurrent health-care systems involving a wide range of health facilities and individual practices.

In TB, the DOTS program is traditionally designed for NTP networks of public services. Evidence however suggested that a significant part of TB patients seek and receive care from the private medical sector as a first resort (42). The first point of contact plays a major role in many respects, including early diagnosis and adherence to treatment, which appeared to be two major challenges in TB control. More than a production of care services, health systems should support ill people in realizing their potential health. Therefore, reaching continuity of care is crucial as transition points between services or quality issues mostly occur at the boundaries of services (43). Yet, the success of many health programs is undermined by poor functioning and fragmented primary health-care provision. Recognition of a medical pluralism may then improve equitable access to care and health outcomes. Hence, the first-line health stakeholders tend to be critical pieces of research knowledge translation into action and increased research impact on policy and practice (44).

Private care providers have been substantially involved to improve TB case notification and patient treatment (45, 46). Yet, inappropriate TB management practices of for-profit practitioners were reported in various settings, e.g., use of chest X-rays for diagnosis instead of sputum smear microscopies, irrational drug prescriptions (47). The use of quality care services by the poor was also a core issue in the interrogation of private care delivery (48). In response, public–private initiatives emerged as a mean to address the epidemics of multi-drug and extensively drug resistant (49) and improve health outcomes (50).

Engaging private stakeholders in TB control was endorsed by the WHO as a global approach toward all-patients management according to national guidelines (51). The End TB strategy aims to promote access to quality high-priority care and better respond to national and local critical issues. In sum, engaging all care providers through public–private mix (PPM) initiatives became a core component of the Stop TB Partnership global strategy a decade ago (52). However, the post-2015 era should still bring the answers to the global challenges of effective engagement of PPMs, efficient use of limited resources, and sustainability (53).

### Opportunities and Pitfalls of Using Collaborative Approaches to Deliver Holistic Health Impact

To make the TB eradication strategy successful, for-profit practitioners involved in PPMs must not only be well trained to taskwork, which likely refers to the execution of the DOTS (54) but also to teamwork, which refers to interactive behaviors to foster team performance (55). Yet, issues related to role division and collaboration modes were identified as weak components of contracting processes where PPMs were initially implemented (56). Several barriers to effective teamwork may persist, including poor adaptability to changes and seamless dialog between all parties (56). A series of bottlenecks to teamwork were raised in the literature, both within the public and private sectors. They covered inadequate training to collaborative work, absence of operative regulations, perceived little common ground for teamwork, low quality of care, and even reluctance from private providers with respect to “loosing” patients (42); peer influence, care-seeking attitudes, and cultural aspects (46). These aspects mostly refer the XX′ policy gap because of the focus on the supply of care services. To simplify, we opted to emphasize the most direct links with these different aspects and policy gaps. However, provision of care cannot be dissociated with other aspects such as the responsiveness of services offered with respect to beneficiaries’ needs (e.g., the need for social support and pre-payments or pooled schemes) (cf. the YY′ and ZZ′ gaps). The systemic reasoning must take precedence over a tunnel vision.

Increased synergy between sub-sectors without burdening public resources remains a crucial issue for many resource-poor countries. Concurrently, positive gains for patients are expected (cf. the ZZ′ gap). So far, most impact measurements of PPMs relied on program-based or medico-focused process and outcome indicators, often neglecting to assess potential gains in financial health protection. Nonetheless, the integrated approach—limited to PPMs in this case—intends to address the economic burden of disease.

Among the first to evaluate PPMs from an economic perspective, an Indian study revealed major drivers of patient costs before TB diagnosis and during treatment, respectively, lost wages and non-medical expenditure on transport (57). For high-burden patients, significant improvement in financial protection and access to quality care were attributed to PPMs. Globally, PPMs were indeed promised as cost-effective approaches to foster equity in TB care access and financial protection for the poorest (51). Recently, such partnerships allowed to lower direct and indirect costs for patients treated in PPM programs versus non-PPM sector, up to five times less for out-of-pocket expenditure and half as much for lost income (58). Particularly, PPM considerably lowered the burden of transportation costs to access TB services (46). Recent evidence on cost savings opportunities for TB patients using PPMs thus confirmed their potential effective contribution to achieve the poverty-related aspirational goal of reducing financial burden of illness (i.e., the newly promoted End TB Goal).

By contrast, other studies highlighted additional consultation fees or spending on diagnostic tests, drugs for complications or herbal medicines in PPMs (cf. the XX′ gap). Recall that these elements were featured as major contributors to the economic burden borne by TB-affected households (the ZZ′ gap). Besides, although pointed as cost-effective at short term in high-burden countries (59, 60), it should be raised that some PPMs require running under substantial investments (notably for orientation and referral procedures sensitization tools and training activities). Moreover, their outcomes varied significantly according to local care delivery settings and contextual factors (58). Then, new evidence is needed to first, better document budget impact of potential extra fees charged for consultation, diagnostic tests, or TB medicines to manage TB and coinfected cases, and second, prioritize services by cost-effectiveness in order to avoid forgoing of potential large gains for patients and health programs. Engaging various actors in PPMs thus impose to deal with complexity. Future research is expected to actually conclude on cost-effectiveness of PPMs on the long run.

Obviously, there should be numerous innovative ways of achieving the three strategic End TB goals simultaneously. One can be through applying effective collaborative work with the specific aim of improving programmatic performance for TB control together with the patients’ perspective (cf. the X′Y′Z′ policy gaps). Showing successful NTP outcomes attributed to a reorganization of the work into a collaborative approach, a “Quality Assurance Project” implemented in rural Bolivia caught our attention (61). The core process was to set up a series of quality improvement teams conducted by collaborative leaders and composed by health workers and NTP regional and national stakeholders. Then, engaged first-line practitioners raised awareness on the benefits of changes in both patients’ and providers’ behaviors toward improved care use and delivery. Comprehensive measurement guidelines to assess collaborative patterns among all first-line health workers should help reaching evidence-based decision-making. In Bolivia, the focus given on quality performance of collaborative work allowed developing the most appropriate solutions to address the locally identified programmatic gaps and clinical problems. Indeed, the use of monitoring and evaluation indicators was promoted and related to low-performance issues such as the lack of compliance to treatment and early detection, limited DOTS practice in rural areas, weak drug logistics, poor lab quality control, and deficiency of clinical training of health staff—of which most were reported in our case studies. Key concerns in this dynamic would rely in producing the development of relevant knowledge and effective data use for local and programmatic improvements. A sector-wide implementation approach to comprehensive assessment of PPMs would yet serve to better predict catastrophic health expenditure incurred by TB patients as well as local breaches and actors who need to work collaboratively toward improved seek, use, and delivery of care services (37). Getting the right information will definitely help to produce the best value in health.

Heavy reliance on out-of-pocket payments for health, with possibly a large portion of it spent within the private sector calls for utmost caution. Evidence must support the added value of inclusive approaches in terms of positive actions such as improving the quality of patients’ care pathways and reducing the associated economic burden of illness. At the same time, it is essential to take account of the risks potentially induced by policies aimed at strengthening or expanding the private sector or its role in public provision of health-care services. A controversial report highlighted several weaknesses of the mainstream optimism in favor of commercial private health-care delivery in poor countries (62). In this respect, the recommendations may include to maintain interest in growth and support in the public sector, to strengthen the evidence that can demonstrate the societal benefits of PPPs, and the importance of capacity building to better regulate health provision.

## Conclusion

This study brings to the fore that low-income households tend to be hindered from accessing primary care services delivered within pro-poor systems due to a complex pattern of interrelated financial and weak system reasons. It provides critical clues on supply induced catastrophic health expenditure mostly due to a lack of responsiveness to local needs in the implementation of national health programs. Out-of-pocket payments made at the point of services are central in health financial reforms toward increased financial protection and UHC, which are currently operated in many countries over the world. Those countries experience serious challenges for offering and maintaining delivery of quality health care for all.

Comprehensive assessment of the economic burden of illness may inform health planners and decision-makers with the development goals. Used as a lever of change for this purpose, the poverty disease focus sounds promising. The TB control strategy was based on strong scientific evidence. Vertical disease-specific programs such as TB control may have non-negligible positive effects on the accuracy and completion of data collection in monitoring reports. Realizing transversal integration with the silos of health programs such as TB or HIV/AIDS and other medical and social care services can make complex health systems better responsive to the crucial issues of multi-morbidity, erratic care pathways, and economic burden of illness. Congruent and collaborative practices of multiple health entities and practitioners, including private actors and patients themselves, are challenging but needed for holistic health impact and case management optimization.

To conclude, the full spectrum of possible interventions to facilitate cost-effective PPMs would include different approaches i.e., the implementation of locally relevant tools and guidelines for practice, benchmarking practices, taskwork and teamwork training, learning projects to build mutual confidence between parts, early participation of coordinated stakeholders, actors’ involvement in planning, process facilitator entities, contracting, and regulation. Joint efforts of the existing health-care sub-sectors, private and public, tend to constitute a way to solve underperforming TB control activities provided in low-income settings. Nevertheless, a common concern of public health workers remains the need to accommodate with the patients’ environment and patterns to ensure readiness of and compliance to health programs activities. Funding mechanisms should support collaborative work practices to integrated care delivery and stakeholders’ engagement. Additionally, integrated care opportunities should remain flexible to providers co-developed guidelines and other instruments for patient-centered care. These must address local priorities and all health actors’ new needs. Rational decisions for efficient and equity-friendly disease-specific control interventions will rely on a transparent evidence base that can be provided through regular assessment and monitoring of policy responsiveness to improved health goals. Various high-level meetings highlighted the importance of measuring the impact of PPMs, both in terms of cost-effectiveness and ability to reduce economic burden of disease (53, 63). Then, comparative patient costs studies and cost-effectiveness evaluations are necessary to build strong and informed health policies. This calls for innovative forms of effective partnerships not only to care but to prevention and control of TB and other illnesses.

## Author Contributions

SL is the only author of this work, executing all research, manuscript, and figure preparation.

## Conflict of Interest Statement

The author declares that the research was conducted in the absence of any commercial or financial relationships that could be construed as a potential conflict of interest.

## Footnotes

^1^“Human choices and ingenuity worn by social, political, and economic constructs.”
